# Transcriptional regulation of *CYP1*9 by cohesin-mediated chromosome tethering in human granulosa cells

**DOI:** 10.1016/j.bbrep.2021.101086

**Published:** 2021-07-24

**Authors:** Naoe Kotomura, Nobuhiro Harada, Yohei Shimono, Satoru Ishihara

**Affiliations:** Fujita Health University School of Medicine, Toyoake, Aichi, 470-1192, Japan

**Keywords:** Chromatin, Promoter, Enhancer, Silencer, TBP, Aromatase

## Abstract

Human *CYP19* spans a region of chromosome 15 of approximately 130 kb and encodes aromatase, an enzyme required for estrogen synthesis. In the human granulosa cell-line KGN, there are seven open chromatin regions within the *CYP19* locus. In this study, we demonstrate that two of these regions ~40 kb upstream and ~15 kb downstream of the *CYP19* promoter are cohesin-loading sites, physically interacting with the promoter to negatively and positively regulate transcription, respectively. These observations suggest that *CYP19* expression is controlled by a balance between the upstream silencer and downstream enhancer. When cohesin is depleted, *CYP19* expression is elevated since the silencer is 2.5-fold further from the promoter than the enhancer and most likely depends on cohesin-mediated tethering to influence expression.

## Highlights

• Silencer and enhancer elements of *CYP19* were identified in the human granulosa cell-line KGN.
• The silencer and enhancer elements both interacted with the *CYP19* promoter through cohesin-mediated chromosome tethering.
• A balance between the activity of the silencer and enhancer element controls *CYP19* expression.

## Abbreviation

3C: chromosome conformation capture
cE4: a core region of EUS-4
cE7: a core region of EUS-7
ChIP: chromatin immunoprecipitation
EUS: enrichment in upper fractions of the SEVENS assay
GTF: general transcription factor
H3K27ac: acetylation of histone H3 lysine 27
luc: luciferase
RNAP: RNA polymerase
SE: standard error
SEVENS: sedimentation velocity centrifugation followed by normalization in the size of the DNA
SMC: structural maintenance of chromosomes
TF: transcription factor

## 1 Introduction

*CYP19* encodes aromatase, which catalyzes the conversion of androgen to estrogen, and is transcribed in several steroidogenic tissues, including placental, gonadal, and adipose tissue [1]. Human *CYP19* resides in a region of chromosome 15 of approximately 130 kb, and is comprised of multiple non-coding exons and nine common coding exons [1]. Multiple promotors of *CYP19* are flanked by non-coding exons and promotor utilization is tissue-dependent: promoter 1a, also referred to as I.1, is active in the placenta; promoter 1b, also referred to as I.4, is active in adipose tissue and the fetal liver; and promoter 1c, also referred to as I.3 or PII, is active in the ovary [2]. In ovarian follicle maturation, immature granulosa cells differentiate into mural granulosa cells, which compose follicle walls, and cumulus cells, which surround and maintain oocytes [3,4]. *CYP19* transcription occurs from the 1c promoter in mural granulosa but not cumulus cells, and the transcription begins at antral follicles [2,5,6]. To study the spatial and temporal regulation of *CYP19* in granulosa cells, the human ovarian granulosa tumor cell-line KGN can be used [7].

Following improvements in chromatin dissection methods, the relationship between transcriptional regulation and chromatin structure has become better understood. We have developed the SEVENS (sedimentation velocity centrifugation followed by normalization in the size of the DNA) assay, a method for the fractionation of chromatin by degree of compaction [8]. Using this method, we found seven open chromatin regions within the *CYP19* locus in KGN cells, which were designated EUS-1 to EUS-7 (enrichment in upper fractions of the SEVENS assay) [9]. Open chromatin is usually found in regions containing active promoters and enhancers, to which RNA polymerase (RNAP) and transcription factors (TFs) bind, respectively [10]. Thus, we hypothesized that EUSs function as regulatory elements for *CYP19* transcription in KGN cells. Some regulatory elements, such as enhancers, are located far from their target promoter. The functions of such distal elements require the formation of chromatin loops to facilitate their physical interactions with promoters [11]. Cohesin is a ring-shaped protein complex that consists of two SMC (structural maintenance of chromosomes) proteins, SMC1 and SMC3, and other accessory proteins, and is loaded onto regulatory element-promoter interaction sites, where a cohesin ring strangles the neck of chromatin loops, tethering two regions separated by the loop [12,13]. Together with the observed colocalization of cohesin with transcription coactivators [14], assessment of cohesin recruitment is valuable for studying *CYP19* EUSs as potential regulatory elements.

In this study, we identified EUS-4 and EUS-7 as regions of SMC1 recruitment and found that they interacted with the 1c promoter through cohesin-mediated chromosome tethering. Reporter assays indicate that EUS-4 operates negatively on the promoter, while EUS-7 has a positive effect, suggesting that *CYP19* transcription in KGN cells is determined through a balance of these opposing activities. When these interactions are disrupted upon the depletion of cohesin, *CYP19* transcription is elevated, suggesting that the negative effect of EUS-4 is affected. EUS-4 is 2.5-fold further from the 1c promoter than EUS-7 and the activity of EUS-4 over the *CYP19* promoter is, therefore, more likely to be dependent on chromosome tethering.

## 2 Materials and Methods

### 2.1 Cell culture

KGN cells were provided by RIKEN BRC through the National Bio-Resource Project of the MEXT, Japan, and were cultured in Dulbecco's Modified Eagle Medium: Nutrient Mixture F-12 supplemented with 10 % fetal bovine serum and antibiotics. SMC1-targeting siRNAs (#L-006833-00-0005) and non-targeting control (#D-001810-10-05) were purchased from Dharmacon. siRNAs were transfected into KGN cells using DharmaFECT (#T-2005, Dharmacon) following the manufacturer's instructions. Cells were harvested for analyses 5 days after transfection.

### 2.2 Chromatin immunoprecipitation (ChIP), western blotting, and RT-PCR analyses

ChIP, western blotting, and RT-PCR analyses were performed as described previously [9]. Primary antibodies for ChIP and western blotting were as follows: anti-SMC1 (#A300-055A, Bethyl Laboratories), anti-histone H3 (#ab1791, Abcam), anti-H3K27ac (#39685, Active Motif), anti-c-Jun (#sc-44X, Santa Cruz Biotechnology), anti-TBP (#sc-273X, Santa Cruz Biotechnology), anti-RNA polymerase II (#sc-899X, Santa Cruz Biotechnology), anti-TFIIB (#sc-225X, Santa Cruz Biotechnology), and anti-β-actin (#A1978, Sigma-Aldrich). PCR primers for ChIP and RT-PCR are listed in the Supplementary Materials.

### 2.3 Chromosome conformation capture (3C) assays

KGN cells in a 10-cm dish were incubated in a fresh culture medium with 1 % formaldehyde at room temperature for 10 min before glycine was added at 125 mM to quench the formaldehyde. Cells were washed twice with PBS, harvested, and resuspended in 1 ml of ice-cold Nuclei Preparation Buffer (10 mM Tris-HCl (pH 7.5), 10 mM NaCl, 0.2 % NP-40, Complete protease inhibitor cocktail (Roche)). The cell suspension was homogenized with 20 strokes of a Dounce homogenizer (with tight clearance) on ice and agitated at 4 °C for 1 h. Following centrifugation at 3445×*g* for 5 min, 5 mg (wet weight) of nuclei were resuspended in 100 μl of 1x NEBuffer 2 (#B7002, New England BioLabs) supplemented with 0.3 % SDS and agitated at 37 °C for 1 h. Nuclei were mixed with 10 μl of 20 % Triton X-100, agitated at 37 °C for an additional 1 h, and incubated with 1000 units of HindIII (#R3104, New England BioLabs) at 37 °C overnight. Digested nuclei were treated with 20 μl of 10 % SDS at 65 °C for 20 min to inactivate HindIII and divided into two aliquots: the first aliquot was diluted with 1330 μl of 1x ligase reaction buffer (#B0202, New England BioLabs) supplemented with 1 % Triton X-100, incubated at 37 °C for 1 h, and incubated with 4000 units of T4 DNA ligase (#M0202 M, New England BioLabs) at 16 °C for 20 min; the second aliquot was similarly processed but T4 DNA ligase was omitted. After crosslink reversal, DNA was recovered from the nuclei using ethanol precipitation. One twelfth of the DNA preparations were applied to a single PCR reaction. As a control for ligation products, a 175 Mb BAC clone that covers the entire *CYP19* gene (clone ID: RP11–184P8, BACPAC Genomics) was digested by HindIII and then ligated with T4 DNA ligase randomly. Ligated BAC (90 fg) was applied to a single PCR reaction. PCR was performed as follows: 32 cycles of 95 °C for 30 s, 65 °C for 30 s, and 72 °C for 30 s. PCR products were analyzed by agarose gel electrophoresis. PCR primers are listed in the Supplementary Materials.

### 2.4 Luciferase (luc) assays

KGN cells were seeded at 1 × 10^5^ per well in a 24-well plate 1 day before transfection. One hundred nanograms of pNL1.1[*Nluc*] (#N1001, Promega)-based Nanoluc reporter constructs (described below), 5 ng of pGL4.13[*luc2*/SV40] firefly luciferase reporter control plasmid (#E6681, Promega), and 395 ng of pBluescript-SK (−) as a carrier were transfected into KGN cells using Lipofectamine 3000 (#L3000001, Thermo). Two days after transfection, the cells were lysed in Glo lysis buffer (#E2661, Promega) and divided into two aliquots. Using the Nano-Glo Luciferase assay system (#N1120, Promega), luminescence resulting from Nanoluc and firefly luciferase were measured separately in an ARVO X multilabel reader (PerkinElmer). Nanoluc activity is expressed as a proportion of firefly luminescence. A series of reporter constructs were created using pNL1.1[*Nluc*], into which fragments of the *CYP19* gene were inserted at the position shown in Fig. 3A–C. The fragments used were as follows: the 1c promoter, 100119–100571; cE4, 61525–62534; cE7, 114605–115632; cE7a, 114605–115137; cE7b, 114878–115312; cE7c, 115138–115632; cE7ab, 114878–115154 (in RefSeq NG_007982).

## 3 Results

### 3.1 Tethering between the CYP19 promoter and open chromatin regions by cohesin

**Fig. 1:**
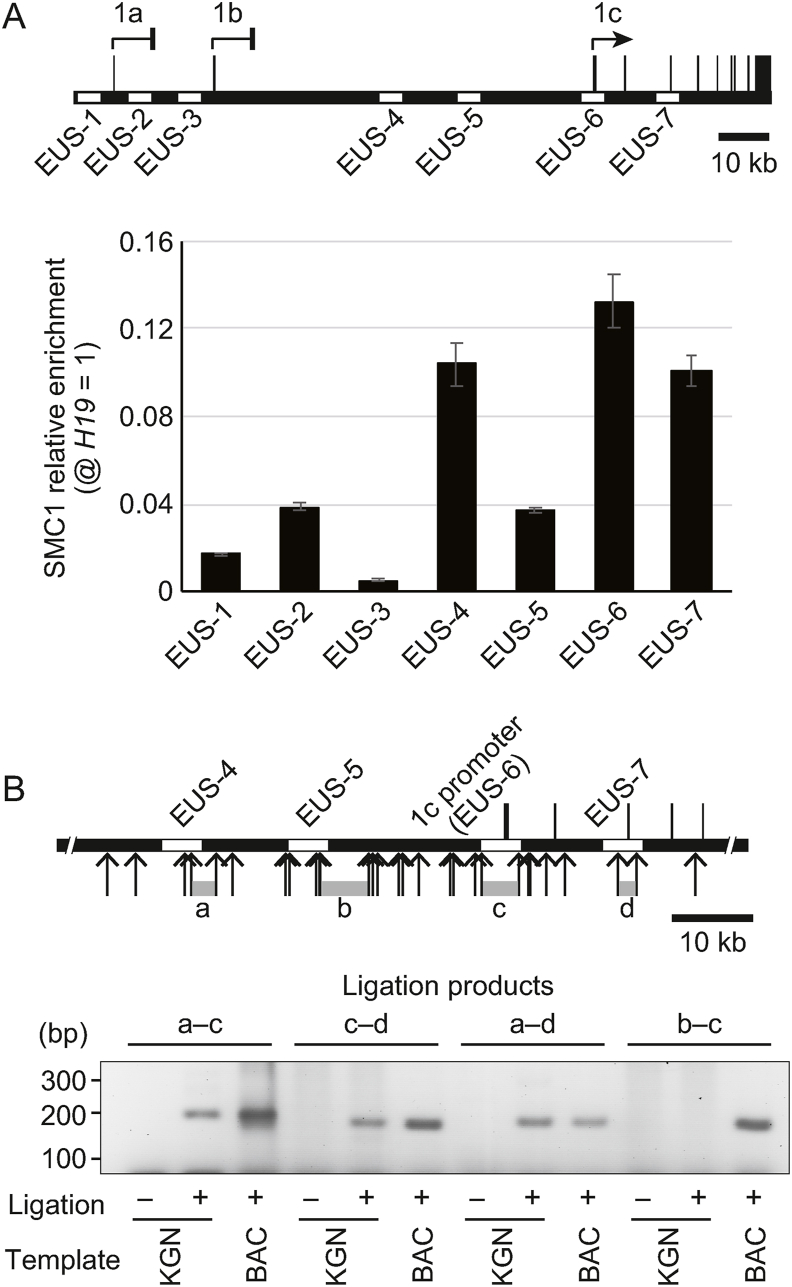
Interaction between the 1c promoter and the open chromatin regions EUS-4 and EUS-7 by cohesin-mediated tethering. (A) The upper panel shows the entire *CYP19* locus in which seven “EUS” open chromatin regions in KGN cells are represented as open rectangles. *CYP19* transcripts in KGN cells are from the 1c promoter alone (marked with an arrow), and not the 1a or 1b promoter (marked with short bars). The lower panel shows the relative enrichment of SMC1 at each EUS compared with the *H19* locus. Data is represented as the mean ± standard error (SE), which was calculated from at least three independent experiments. **(B)** The upper panel shows a magnified view of the *CYP19* locus, in which EUS-4, EUS-5, the 1c promoter (EUS-6), and EUS-7 are represented as open rectangles. Arrows indicate HindIII restriction sites for 3C assays. Ligation of the HindIII fragments marked with gray bars labeled a to d were analyzed. KGN nuclei and a *CYP1*9 BAC clone were examined using the 3C assay. Representative PCR products are shown in the lower panel. As a control, KGN nuclei were also processed without ligation.

In our previous study, the seven open chromatin regions EUS-1 to EUS-7 were identified within the *CYP19* gene in human ovarian granulosa KGN cells (Fig. 1A) [9]. EUS-6 corresponds to the promoter active in KGN cells, promoter 1c. Other EUSs were thought to be regulatory elements for *CYP19* transcription. To investigate whether these EUSs physically interact with 1c through chromosome tethering, we performed ChIP assays to analyze recruitment of SMC1, a component of cohesin tethering complexes. SMC1 bound to EUS-4, EUS-7, and the 1c promoter region, suggesting that cohesin has the potential to bind EUS-4 and EUS-7 to the 1c promoter (Fig. 1A). To investigate whether these regulatory elements physically interact, 3C assays, in which formaldehyde-treated chromatin was subjected to HindIII digestion and subsequent ligation, were performed. A HindIII fragment containing the 1c promoter was ligated with fragments of EUS-4 and EUS-7 (“a–c” and “c–d”, respectively, in Fig. 1B), but it failed to ligate with EUS-5 lacking SMC1 (“b–c” in Fig. 1B), indicating that EUS-4 and EUS-7, but not EUS-5, were in close proximity to the 1c promoter. Interestingly, a ligation product between EUS-4 and EUS-7 was also detected (“a–d” in Fig. 1B). These observations suggest that EUS-4 and EUS-7 both interact with the 1c promoter, and furthermore interact with each other.

### 3.2 Biased distribution of nucleosomes within EUS-4 and EUS-7

**Fig. 2:**
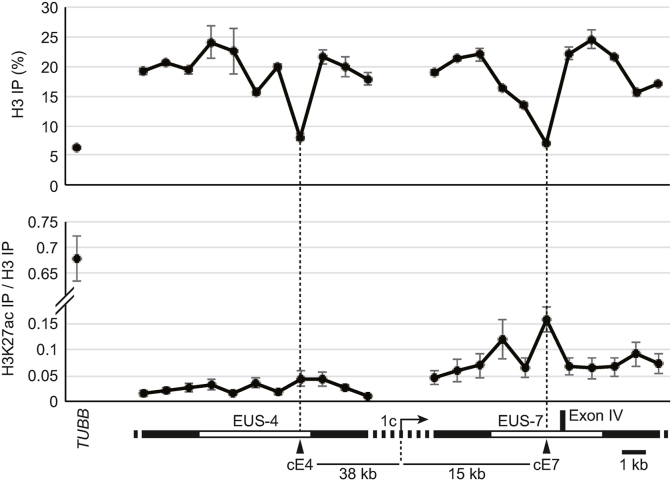
Distribution of nucleosomes and H3K27ac in EUS-4 and EUS-7. ChIP assays using an anti-pan histone H3 and an anti-H3K27ac antibody revealed the distribution of total H3 and H3K27ac across EUS-4 and EUS-7. The nucleosome occupancy is shown in the upper chart as total H3 precipitation percentages. The H3K27ac level is expressed in the lower chart as precipitation percentages normalized to total H3 precipitation. A magnified view of EUS-4 to EUS-7 is shown below the x-axis label. The positions with lowest nucleosome occupancy within EUS-4 and EUS-7 are marked with arrowheads and labeled “cE4” and “cE7”, respectively. The distances between these sites and the 1c promoter are also shown. The *TUBB* promoter was analyzed as a control for open chromatin. Data represent the mean ± SE calculated from at least three independent experiments.

Large protein complexes of TFs and co-factors are recruited to transcription regulatory elements, in consort with the removal of nucleosomes [10]. To clarify whether EUS-4 and EUS-7 function as such elements, we performed ChIP assays using an anti-histone H3 antibody to assess the distribution of histone H3 every 1 kb within the EUSs. H3 was reduced at regions 38 kb upstream (in EUS-4) and 15 kb downstream (in EUS-7) of the 1c promoter (Fig. 2), indicating reduced occupancy of nucleosomes. This was comparable to the promoter region of *TUBB,* which is abundantly transcribed in KGN cells. Acetylation of histone H3 lysine 27 (H3K27ac), an epigenetic marker for open chromatin [15], at both sites was much reduced compared with the *TUBB* promoter (Fig. 2). This suggests that open chromatin at these sites is likely to form independently of H3K27ac. These nucleosomeless sites in EUS-4 and EUS-7, designated as cE4 (core of EUS-4) and cE7 (core of EUS-7) elements, respectively, were hypothesized to function as regulatory elements for *CYP19* transcription in KGN cells.

### 3.3 Effects of the cE4 and cE7 elements on transcription of the CYP19 gene

**Fig. 3:**
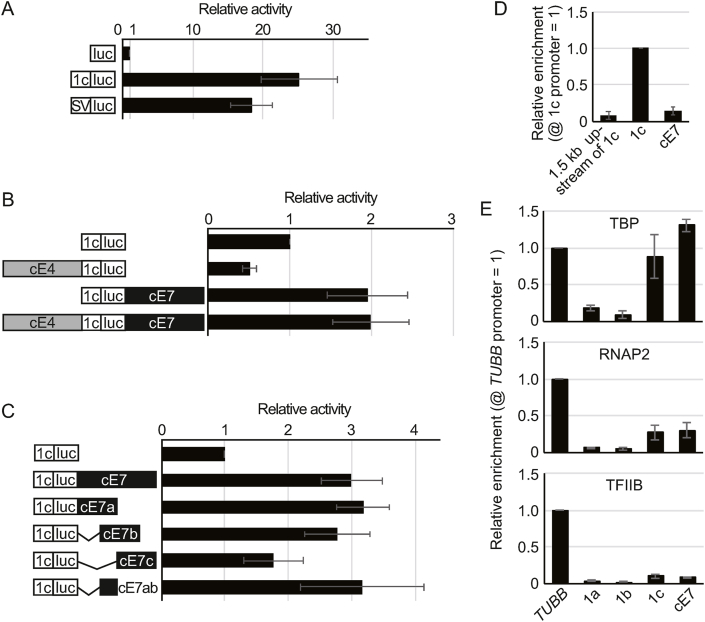
The activity of cE4 and cE7 elements in the regulation of *CYP19* transcription. (A) Basal activity of the 1c promoter was measured in comparison with the SV40 promoter using luc assays. Activity was calculated relative to a promoter-less construct. **(B)** Constructs linking a 1c-luc unit to cE4, cE7, or both were analyzed. Activity was calculated relative to the 1c-luc construct. **(C)** Luc constructs with a series of cE7 truncations were analyzed. Activity was calculated relative to a construct with the1c-luc construct. The assay series in (A), (B), and (C) were conducted separately. **(D)** ChIP assays with anti-c-Jun showing recruitment of c-Jun to *CYP19* 1c promoter and cE7. A region 1.5 kb upstream of the promoter was also analyzed as a negative control. Bars represent mean relative enrichment compared with the 1c promoter ± SE from at least three independent experiments. **(E)** ChIP assays with anti-TBP showing recruitment of TBP to *TUBB*, *CYP19* promoters 1a, 1b, and 1c, and cE7 (upper chart). In the middle and lower charts, the recruitment of RNAP2 and TFIIB, respectively, to these regions was similarly examined by ChIP. Bars represent mean relative enrichment compared with the *TUBB* promoter ± SE from at least three independent experiments.

To evaluate whether cE4 and cE7 elements contribute to transcription of *CYP19*, luc assays were performed in KGN cells. We first examined the transcription activity of a 453 bp fragment that covers the 1c promoter (see Materials and Methods). The 1c promoter largely enhanced luc transcription compared with a control construct, and this enhancement was comparable to the activity of the SV40 promoter (Fig. 3A). Next, we investigated the effects of cE4 and cE7 elements on 1c promoter activity. Fragments of approximately 1000 bp that included either cE4 or cE7 were inserted upstream or downstream, respectively, of the 1c-luc construct according to their native position in the *CYP19* gene. The luc activity of the cE4-containing construct was half that of the promoter alone construct, while luc activity of the cE7 construct was clearly higher than that of the promoter alone (Fig. 3B). These observations suggest that cE4 and cE7 function as a silencer and enhancer, respectively, of the 1c promoter. Interestingly, a construct including both the elements had luc activity comparable with the cE7 construct (Fig. 3B), suggesting that the enhancer activity of cE7 is dominant over the repressive effect of cE4.

### 3.4 Binding of TBP to the cE7 element

To further characterize the enhancer activity of cE7, truncated fragments were integrated into the reporter construct (see Materials and Methods) and tested in luc assays. cE7a and cE7b elements had luc activity indistinguishable from the entire cE7, while the activity of cE7c was significantly reduced (Fig. 3C). A construct containing a 277 bp region overlapping cE7a and cE7b (cE7ab) displayed luc activity comparable with cE7a, cE7b, or the entire cE7 sequence (Fig. 3C). These observations suggest that the enhancer activity is located within a sequence within cE7ab. When a search for TFs bound to cE7ab was conducted using PROMO [16,17], AP-1 and TFIID were identified. ChIP with an antibody against the AP-1 subunit c-Jun, which is recruited to the 1c promoter in KGN cells [18], failed to precipitate cE7 (Fig. 3D). However, ChIP with an antibody against TBP, a component of the TFIID complex, precipitated the cE7 element at a level comparable to the *TUBB* promoter (Fig. 3E). TBP is categorized as a general transcription factor (GTF), recruited to promoter regions by and facilitating the activity of RNAP2 and other TFII series GTFs [19,20]. Therefore, we next performed ChIP assays with anti-RNAP2 and anti-TFIIB antibodies. Binding of RNAP2 and TFIIB to cE7 was comparable with binding to the 1c promoter but was lower than to the *TUBB* promoter (Fig. 3E). These observations suggest that TBP is recruited to cE7 independent of RNAP2 and other components of TFII complexes.

### 3.5 The negative effect of cE4 on transcription from the 1c promoter depends on SMC1-mediated tethering

**Fig. 4:**
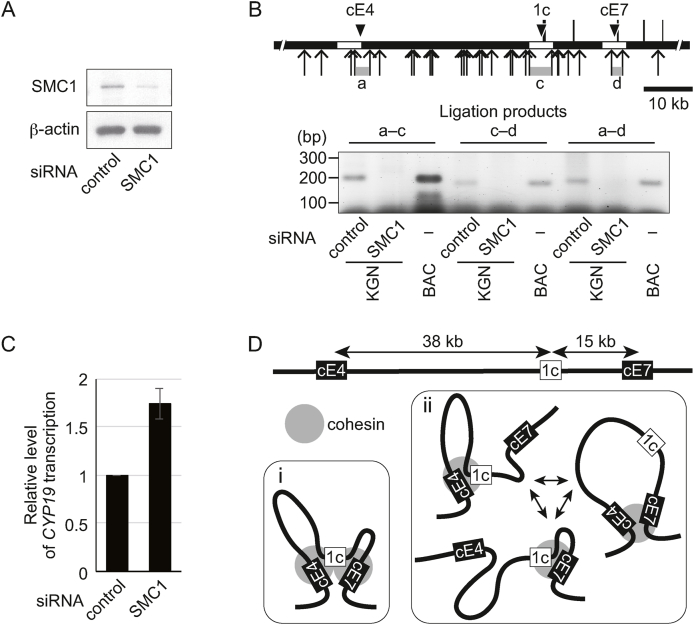
Disruption of chromosome tethering elevates *CYP19* transcription. (A) Using western blotting, the protein levels of SMC1 and β-actin was examined in KGN cells treated with SMC1-targeting siRNA or non-targeting control siRNA. **(B)** The upper panel shows a magnified view of the *CYP19* locus with cE4, cE7, and the 1c promoter (solid black triangles). Arrows indicate HindIII restriction sites for 3C assays. Ligation of HindIII fragments marked with gray bars labeled a, c, and d were analyzed. The lower panel shows representative PCR products from the 3C assay. A *CYP1*9 BAC clone was used as a control. **(C)** RT-PCR analyses showing that treatment with SMC1 siRNA results in an increase in *CYP19* expression. Bars represent the mean ± SE from at least three independent experiments. **(D)** Two alternative models for the regulation of *CYP19* transcription by cohesin-mediated tethering. Multiple 3C signals between either two of cE4, cE7, and the 1c promoter indicate the presence of different interaction combinations in the cell population tested. This suggests two possibilities: (i) ternary interaction occurs; (ii) either two of cE4, cE7, and the 1c promoter interact, but the interaction pair changes constantly.

To examine whether tethering by cohesin is required for the effect of cE4 and cE7 elements on transcription from the 1c promoter, SMC1 was depleted in KGN cells by siRNA. SMC1 protein level was significantly reduced compared with treatment with a control siRNA (Fig. 4A), suggesting impairment of cohesin formation. 3C assays showed that neither cE4 nor cE7 interacted with 1c promoter in SMC1-depleted cells (Fig. 4B). Interestingly, transcription of *CYP19* increased 1.7-fold upon disruption of SMC1 (Fig. 4C). These data suggest that the negative regulatory element cE4 requires cohesin tethering to disrupt *CYP19* expression. The distance between cE4 and the promoter is 2.5-fold greater than between cE7 and the promoter (Fig. 4D), and it is possible that the regulatory effect of cE4 may depend on SMC1-mediated tethering more critically.

## 4 Discussion

In this study, we have identified three cohesin-loaded regions containing the cE4 silencer, the cE7 enhancer, or the 1c promoter within the *CYP19* locus (Fig. 1A). 3C assays using KGN cells indicated that cE4 and cE7 both associate with, and become tethered to, the 1c promoter (Fig. 1B). Because the 3C data were obtained from multiple cells, how these elements interact, and the structure of these interactions, in individual cells, is unclear. Considering the homogenous expression of the *CYP19* gene in KGN cells [7], it is possible that either: (i) a ternary complex of cohesin sites forms, creating two loops, uniformly; or (ii) either two of the cohesin sites become tethered with a single loop, with the interaction pair changing constantly (Fig. 4D). Luc reporter constructs linking cE4 and cE7 side-by-side imitated this ternary complex but showed comparable activity to constructs with cE7 alone (Fig. 3B). This suggests that the positive effect of cE7 may be, in part, dominant over the negative effect of cE4. However, when KGN cells were treated with siRNA against SMC1, *CYP19* transcription was upregulated (Fig. 4C), suggesting the disruption of any negative regulation by cE4. The distance between the 1c promoter and cE4 or cE7 is 38 kb or 15 kb, respectively (Fig. 4D). The dependency on cohesin-mediated tethering by cE4 may result from this greater distance between this element and the 1c promoter, while it is possible that the cE7 interaction with the 1c promoter may be stable without cohesin tethering. We identified TBP as a TF that binds to cE7 (Fig. 3E). Although TBP is well characterized as a GTF, the distribution of TBP in the genome is different from other GTFs [21]. We observed that TBP, but not the GTFs RNAP2 or TFIIB, was recruited to cE7 (Fig. 3E), consistent with this unique distribution of TBP. Considering that TBP is capable of interacting with the coactivator SAGA [22], it is possible that TBP binding to cE7 may function as a component of this coactivator. *CYP19* is transcribed at a lower level in KGN cells than other steroidogenic cells [9], implying that *CYP19* may not be fully activated in KGN cells. This moderate expression of *CYP19* is likely included in immature features of KGN cells, which derive from incompletely differentiated cells rather than mural granulosa cells [7]. It is possible that cE4 may control the start of transcription of *CYP19* as a differentiation marker during cellular maturation. Further studies are needed to clarify this temporal regulation in granulosa cells during folliculogenesis.

## Declaration of competing interest

The authors declared that they have no conflicts of interest.

## Funding

This study was supported by the KAKENHI grants from 10.13039/501100001691Japan Society for the Promotion of Science (JSPS), Japan (#16K11118 and #20K09632 to N.K.).
