# Supplementary material for: Transcriptional regulation of CYP19 by cohesin-mediated chromosome tethering in human granulosa cells
Source: Biochem Biophys Rep. 2021 Jul 24;27:101086. doi: 10.1016/j.bbrep.2021.101086 (PMC8326343; doi:10.1016/j.bbrep.2021.101086)
Supplement: Multimedia component 1 [file mmc1.pdf]

Supplementary materials: PCR primers for ChIP, 3C, and RT-PCR analyses

Usage	Figure	*GENE* (Position)	Forward primer	Reverse primer
ChIP	Fig. 1A	*CYP19* (EUS-1)	TCCGAATTGCAACCTATTCC	CAGAGTTAGTGGTAGCACTG
ChIP	Fig. 1A	*CYP19* (EUS-2)	CTGTGGACCCTAAACATGTG	GATACTGGTGCCCTCTTATG
ChIP	Fig. 1A	*CYP19* (EUS-3)	GACACTGAATTTCACAGTGG	GGATTTGGATGTGTCTCTAC
ChIP	Fig. 1A	*CYP19* (EUS-4)	CATAGCCTGAGCTGAAAGAG	TCAAGCAAGCCATTACTCTG
ChIP	Fig. 1A	*CYP19* (EUS-5)	ATCCATCATAGCCTTGCTTG	AGCTATGCCACAGTTTACCC
ChIP	Fig. 1A	*CYP19* (EUS-6)	GACCTCAACGATGCCCAAGA	AAAGGCAATCTCCCAACTCC
ChIP	Fig. 1A	*CYP19* (EUS-7)	AGCTTCCTTTACTACCACAG	GAGTATCAGTCAGGGCTTGA
3C	Fig. 1B	*CYP19* ("a" fragment)	GGAGAAATGCAGGAAGGAGGTGTGTTATGG	-
3C	Fig. 1B	*CYP19* ("b" fragment)	-	GCTCCAGAAACATAGCAAATCTAGCTTCCG
3C	Fig. 1B	*CYP19* ("c" fragment)	-	TTATGGGATTTAGTCCCCACATTCATGGAC
3C	Fig. 1B	*CYP19* ("d" fragment)	-	GCTGCTCTGGTTCAATGTATTAGGTTGTGG
ChIP	Fig. 2	*TUBB* (promoter)	CATTCCAACCTTCCAGCCTG	TTTGCTCGCCTCAAGGTATG
ChIP	Fig. 2	*CYP19* (45 kb upstream of 1c promoter)	CAAAGGGTGAGAGGATGGAG	TCTTGCACCTCAGGTTTCAC
ChIP	Fig. 2	*CYP19* (44 kb upstream of 1c promoter)	AGGAGACAAAGCCTGCATGG	CTGTCTCACATGCTACAAGT
ChIP	Fig. 2	*CYP19* (43 kb upstream of 1c promoter)	CTTGGTAGTCAGGGCACACT	AGTATGGTACCATCAGCCAG
ChIP	Fig. 2	*CYP19* (42 kb upstream of 1c promoter)	GAGCCCTGGACATTATGTCA	ATAATGATAGCTGTGGCGGT
ChIP	Fig. 2	*CYP19* (41 kb upstream of 1c promoter)	CTGGTCCTTCTCCAAGTCGA	ATTCCTGTCAATTTATGGGGA
ChIP	Fig. 2	*CYP19* (40 kb upstream of 1c promoter)	CATAGCCTGAGCTGAAAGAG	TCAAGCAAGCCATTACTCTG
ChIP	Fig. 2	*CYP19* (39 kb upstream of 1c promoter)	CTCTGGAAGCCCAGACTATT	CTGTAGATCATGTGCTTGCC
ChIP	Fig. 2	*CYP19* (38 kb upstream of 1c promoter)	AAGAAGGCGGTTGTGCTTTG	CTGCCTGGAGAAGTCACATC
ChIP	Fig. 2	*CYP19* (37 kb upstream of 1c promoter)	AGTCACCTCCTGATAGGTAG	GGCCATTTGCCTTGGACTCT
ChIP	Fig. 2	*CYP19* (36 kb upstream of 1c promoter)	TTCTTCTGAGCCTTAGCCAC	GAAGACCCACAACCAACTTG
ChIP	Fig. 2	*CYP19* (35 kb upstream of 1c promoter)	ACTGTAAAGTAGCCCCACAA	CATGCACACACATAGATACC
ChIP	Fig. 2	*CYP19* (10 kb downstream of 1c promoter)	ACAGGGGCTTCCTTCATTTA	GGATGAAGAGCCAATAATGAC
ChIP	Fig. 2	*CYP19* (11 kb downstream of 1c promoter)	CATTGAATGGGGACATCGTG	CCACTCAGATGTCAACTGGG
ChIP	Fig. 2	*CYP19* (12 kb downstream of 1c promoter)	TTCCCAGCCCACTTCAACTG	GACCCTCTTCTTACTTTGTC
ChIP	Fig. 2	*CYP19* (13 kb downstream of 1c promoter)	TGCCTCCTCATCACTGTTGA	TCAGGGAGTAAGCTCTTAGG
ChIP	Fig. 2	*CYP19* (14 kb downstream of 1c promoter)	GGAGGGGAAGGTAAGTAAAG	TCTGGGGAATTTGTCTGACG
ChIP	Fig. 2	*CYP19* (15 kb downstream of 1c promoter)	AGCTTCCTTTACTACCACAG	GAGTATCAGTCAGGGCTTGA
ChIP	Fig. 2	*CYP19* (16 kb downstream of 1c promoter)	GAGCTATCATTCCTAGTCTG	TGGCTACAACAGAGTAAAGG
ChIP	Fig. 2	*CYP19* (17 kb downstream of 1c promoter)	CTTCCATGTGCCTGCTGAGA	AAGAGTTGTGCTGAGTCCTG
ChIP	Fig. 2	*CYP19* (18 kb downstream of 1c promoter)	TGCCTATCACTCATGTTGGG	GTCAGATAGGTAGCAAGTGG
ChIP	Fig. 2	*CYP19* (19 kb downstream of 1c promoter)	GGAGGCTGTGGTTTCAAGAA	AGAAACCCTGATCTTGCCTA
ChIP	Fig. 2	*CYP19* (20 kb downstream of 1c promoter)	GGATAGTCTTCTTCTGGAGTT	CTTAAAGTGCCCCTTTCTCT
ChIP	Fig. 3D	*CYP19* (1.5 kb upstream of 1c promoter)	CCAACTGTCAGAAAGTCACA	CTGGTTCCTTTGTTGATGAC
ChIP	Fig. 3D	*CYP19* (1c promoter)	GACCTCAACGATGCCCAAGA	AAAGGCAATCTCCCAACTCC
ChIP	Fig. 3D	*CYP19* (cE7 element)	AGCTTCCTTTACTACCACAG	GAGTATCAGTCAGGGCTTGA
ChIP	Fig. 3E	*TUBB* (promoter)	CATTCCAACCTTCCAGCCTG	TTTGCTCGCCTCAAGGTATG
ChIP	Fig. 3E	*CYP19* (1a promoter)	TTTGCCCTCCTTTCATCCAC	ATTCCTTCCTCCAGGGTATG
ChIP	Fig. 3E	*CYP19* (1b promoter)	GGTTCATCTGTCGTCTTCAG	GAACCACATATTTCCCCAAG
ChIP	Fig. 3E	*CYP19* (1c promoter)	GACCTCAACGATGCCCAAGA	AAAGGCAATCTCCCAACTCC
ChIP	Fig. 3E	*CYP19* (cE7 element)	AGCTTCCTTTACTACCACAG	GAGTATCAGTCAGGGCTTGA
3C	Fig. 4B	*CYP19* ("a" fragment)	GGAGAAATGCAGGAAGGAGGTGTGTTATGG	-
3C	Fig. 4B	*CYP19* ("c" fragment)	-	TTATGGGATTTAGTCCCCACATTCATGGAC
3C	Fig. 4B	*CYP19* ("d" fragment)	-	GCTGCTCTGGTTCAATGTATTAGGTTGTGG
RT-PCR	Fig. 4C	*GAPDH* (Exon IX)	CAGCAAGAGCACAAGAGGAA	CTACATGGCAACTGTGAGGA
RT-PCR	Fig. 4C	*CYP19* (Exon II)	GGAAATGCTGAACCCGATAC	AAAGGCCAGTGAGGAGCAG
